# Medical students’ experiences when empathizing with patients’ emotional issues during a medical interview – a qualitative study

**DOI:** 10.1186/s12909-022-03199-9

**Published:** 2022-03-04

**Authors:** Knut Ørnes Brodahl, Hanne-Lise Eikeland Storøy, Arnstein Finset, Reidar Pedersen

**Affiliations:** 1 Centre for Medical Ethics, University of Oslo, Kirkeveien 166, Fredrik Holsts hus, 0450 Oslo, Norway; 2 Department of Behavioral Sciences in Medicine, University of Oslo, Oslo, Norway

**Keywords:** Medical education, Empathy, Physician-patient relationship, Medical students, Communication, Physicians’ role, Professionalism, Clinical interview, Patients’ emotions

## Abstract

**Background:**

There is evidence that empathy decreases as medical students go through clinical training. However, there are few in-depth studies investigating the students’ own experiences when trying to empathize in concrete clinical encounters. We therefore wanted to explore medical students’ perceptions, experiences, and reflections when empathizing with patients expressing emotional issues.

**Methods:**

A qualitative content analysis of semi-structured interviews with third year medical students (*N* = 11) was conducted using video-stimulated recall from their own medical interview with a simulated chronically ill patient. Students were led to believe that the patient was real.

**Results:**

Five themes which may influence student empathy during history-taking were identified through analysis of interview data: (1) Giving priority to medical history taking, (2) Interpreting the patient’s worry as lack of medical information, (3) Conflict between perspectives, (4) Technical communication skill rather than authentic and heart-felt and (5) The distant professional role.

**Conclusions:**

The participating students described conflicts between a medical agenda, rules and norms for professional conduct and the students’ own judgments when trying to empathize with the patient. To our knowledge, this is the first study ever to document the students’ own perspective in concrete situations as well as how these reported experiences and reflections affect their empathy towards patients. Since we now know more about what is likely to hinder medical students’ empathy, educators should actively encourage group reflection and discussion in order to avoid these negative effects of history taking both inside and outside of the clinical setting.

## Background

Entry into clinical care represents an existential and moral challenge for medical students as they are faced with the suffering of others and have to learn how to deal with the emotional aspect of their work as physicians to be [1]. The main activity in which medical students interact with patients is the medical interview [2]. In the course of a medical interview, students are expected to retrieve medically relevant information while at the same time paying attention to the existential and affective dimensions [2–4]. The ability to demonstrate empathy is internationally recognized as a key clinical skill in medical education and practice [2, 4–6]. Empathy in medicine can be broadly defined as the appropriate understanding and communication of the patient’s experiences and has been reported to encompass cognitive, affective, behavioral, interpretive and moral aspects [7], but controversies still remain as to how empathy in medicine should be defined [8–10].

Despite evidence that self-reported empathy decreases as medical students go through clinical training [11, 12] there are few in-depth studies of students’ own experiences with empathy in medical interviews with patients [9]. Students have been reported to want to form emotional bonds with patients [13] but are ultimately worried about the potential of being overwhelmed emotionally themselves [14] and tend to focus on collecting medical facts [15]. Surprisingly little attention has been paid to the emotional development of students, and to a certain degree medical education today still encourages students to distance themselves from both their own and their patients’ emotions [16, 17]. Some recent qualitative studies on medical students’ empathy have explored students’ perceptions, conceptualizations, or experiences with empathy more generally [18–20]. To our knowledge there are no previous in-depth studies exploring the students’ own perspective on what they actually feel, think or understand when the patient expresses emotional issues during the medical interview, or on what influences the students’ empathy in such concrete clinical encounters. In a recent review of 206 studies on empathy in medical practice, none were reported to look at “concrete details about what the ( …) physician understood/misunderstood” [21]. The aim of the present study was therefore to explore students’ perceptions, experiences and reflections when trying to empathize with patients expressing emotional concerns in a concrete medical interview.

## Methods

**Fig. 1:**
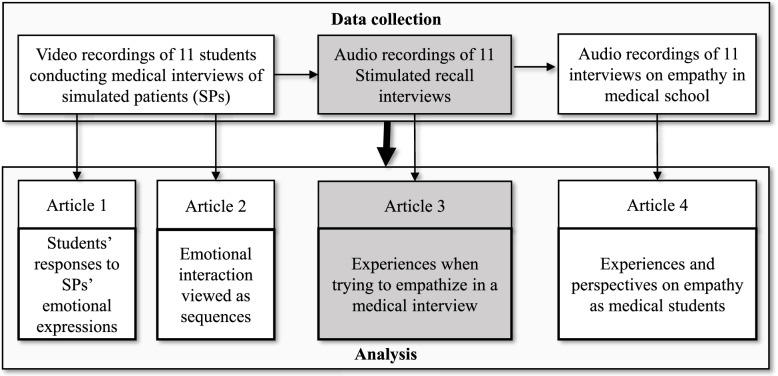
Overview of procedure for data collection and analysis. This paper presented in grey. The complete study included a procedure for data collection in which 11 3rd year medical students were first instructed to conduct a medical interview on a simulated patient while being recorded on video, then interviewed on their perceptions, experiences and reflections when trying to empathize using stimulated video recall (audio recorded) and finally interviewed generally on their experiences and perspectives on empathy in medical school (audio recorded). Article 1, 2 and 4 have already been published (please see present article and reference list for further details)

Data were collected as part of a study in which multiple methods and approaches to data gathering were used to study empathy in medical students in their first year of clinical practice (see Fig. 1). As a part of this study, we conducted a qualitative sub-study with video-stimulated recall interviews to answer the following research question: What characterizes students’ perceptions, experiences and reflections when empathizing with patients expressing emotional issues in a concrete medical interview?

### Participants

We enrolled eleven medical students (six female and five male) as well as four trained female actresses serving as simulated patients (SPs). The strategy employed to recruit the eleven students was purposeful sampling [22]. A total of 19 medical students voluntarily signed up to join the study after a 5-min presentation during a non-mandatory lecture. However, eight of these were not included since we found that the richness of the data with eleven students was sufficient to answer the research questions [23]. All eleven students first completed a medical interview with an SP, before qualitative interviews were conducted by KØB and HES with each student using video stimulated recall (see Fig. 1). None of the participants had any relationship or prior knowledge of KØB and HES’s role in the study other than that they were fellow medical students. Written and voluntary informed consent was obtained from all participants, except that each student got the information that the patient was an SP only after the qualitative interview part of the study was finished. Students were offered a complimentary feed-back session on their clinical communication by a trained communication skills expert in appreciation of their contribution to the study. Since the study was not defined as health research, we were, according to Norwegian regulations, exempted from the obligation to seek approval from the Regional Committee for Medical and Health Research Ethics. The protocol for the research project was approved by the Norwegian Social Science Data Services where aspects of privacy protection were assessed (project number 39888).

### Context

The students, recruited in the second semester of their third year of medical school, had recently learned and practiced clinical skills with the use of role-play, simulated and real patients at a university hospital, such as medical interviewing, physical examination of patients, differential diagnosing, and further patient follow-up. In their second year, all medical students at this institution learnt how to conduct a medical interview which follows a standardized structure in order to gather relevant information as quickly as possible. One requirement during their third year is to independently conduct a minimum of 12 medical interviews and physical examinations of new admissions that are documented as a standardized admission note in the patient’s medical records to be approved by a faculty representative. All of the students had completed prior mandatory courses in communication skills as part of their medical training, including an experiential clinical communication skills course with patients where they practiced “gaining the patient’s perspective” when conducting the medical interview.

### Setting

The study was conducted in spring and autumn 2011 in a communication lab set up to resemble a general practitioner’s office. For each of the medical interviews, the student first received standardized written instructions on her assignment at hand (“The goal of the consultation is to identify the most important features of the patient’s health condition”) as well as a fact sheet on the diagnosis of the patient (an inheritable disease called polycystic kidney disease). We gave the students a task which was very similar to what they would have been given in a typical clinical training situation. Participants were given a time-limit of 20 min to conduct the interview and the mean consultation time was 19 min and 20 s. All consultations were observed on video-link by HES and KØB and recorded on video.

The actors simulated a patient case with polycystic kidney disease from a standardized script developed by KØB, HES, and RP in collaboration with the four actors. The actors were instructed to display emotions related to two problematic situations in the patient’s life: (1) worry attributed to the patient’s insecure future for her and her family resulting in sleep disturbances, and (2) anger/frustration with the father’s primary care physician due to a long delay in the diagnosis of polycystic kidney disease making it impossible for the father to have transplant surgery because of his age and medical condition (and therefore becoming dependent on dialysis for the rest of his life). The actors could freely choose when they would exhibit these emotional concerns (ECs) during the consultation, but were instructed to do so several times, at varying intensities and with verbal as well as non-verbal behavior.

#### Video-stimulated recall interviews

Directly after the end of the consultation the actor reviewed the entire video recording of the consultation accompanied by two members of the researcher group (KØB and HES). The actors were instructed to stop the video where they displayed these ECs, referred to below as EC moments. The actors were asked why they stopped at the particular EC moment, about which scenario they portrayed and how they experienced the communication using a semi-structured interview guide.

Directly after, each student was shown sequences with these ECs (referred to below as EC sequences) from his/her medical interview in a video-stimulated recall interview with both researchers (HES and KØB) using a semi-structured interview guide. The EC video sequences were started approximately 30 s before the EC moment indicated by the actor and ended approximately 30 s after. Some EC sequences included more than one EC moment since several EC moments could be registered within the one-minute EC sequence. Due to time constraints, not all EC moments were shown to students. Instead, the researchers (HES and KØB) would show as many ECs moments as possible and made sure all students viewed at least one EC sequence from each of the two problematic situations. The choice of which EC moments to show students was made by the researchers based on EC moment evocativeness and displayed chronologically in the order they appeared in the video. The number of EC sequences shown to each student ranged from two (containing four EC moments with a total duration of 2 min and 43 s) to seven (containing eleven EC moments with a total duration of 7 min and 23 s).

The EC sequences served as stimuli for recall of the student’s own experience of the events depicted in the videos. Since all 11 students still believed they conducted the medical interview on a real patient during the recall interviews, we will use the term “patient” in the remainder of the article. The students were informed that this procedure was not an examination of any kind, merely an attempt to share thoughts and experiences about interviewing a patient about their health condition. They were further encouraged before each viewing of each of the EC sequences to try to remember what they thought or felt during the medical interview, and not what they thought or felt when reviewing the EC sequence. Students were informed that these sequences were selected because something important had happened or that the patient felt that something important happened in these sequences. For each EC sequence students were interviewed on what they thought the patient was conveying in that particular EC sequence, how the student reacted to what was conveyed, what they thought influenced their response to the patient and how they felt the contact was with the patient. For more information on the students’ verbal behavioral responses to the two different problematic situations, please see [24, 25]. Each of these EC sequence interviews will be referred to as mini interviews in the remainder of the text. The stimulated recall interviews lasted between 25 and 45 min for each student and were recorded on a digital audio-recorder and were transcribed verbatim.

After the video-stimulated recall interviews, the students were interviewed more generally about empathy (see Fig. 1). Results from this last part of the interviews with the students has already been published [20].

### Analysis

To answer the research question, the recall interviews were analyzed based on the principles of qualitative content analysis [26, 27]. The content analysis was mainly conducted by the first author in an iterative process consisting of (1) finding labels or codes for individual student utterances based on interpretations of passages of text using NVIVO 12 software, (2) abstracting meaningful themes that represented higher-order levels of organization of these passages, (3) recoding all passages under these proposed higher-order themes, (4) discussing these proposed higher-order themes in meetings with one of the other authors (RP), and (5) revising these themes multiple times by moving back and forth between steps 1–5. Finally, when RP and KØB had agreed on the themes that were the most representative for the 11 video-stimulated recall interviews, KØB categorized all passages under each respective theme. Passages pertaining to the various themes were then selected and condensed by KØB. Below, the key themes are presented through the sub-titles, while the pertaining content is presented through condensed text and illustrative quotes. Quantifiable terms have been used consistently to give an idea of the numbers of students backing each claim. Generally, “a few” has been used to refer to more than two, “some” between three and five, “most” between six and ten and “all” for all eleven students. This study adheres to the COREQ 32-item checklist for reporting qualitative studies [28].

## Results

The students commented on both the worry and the frustration situations in the recall interviews. All students mentioned both the factual details about the patient’s situation and the patient’s emotional reactions to the events the patient had gone through. When asked about their own experiences during the medical interview however, students often found it difficult to remember or articulate what their own emotional reactions were. Generally, the patient’s situation was described in the recall interviews with more general terms such as “understandable” or “recognizable” and students only occasionally reported having shared the patient’s emotional experience or having felt empathic concern for the patient such as being touched, being moved, feeling sorry for, or experiencing compassion or sympathy. Most students remarked how the patient was easy to talk with and that she was willing to share. Many of the students thought that the patient’s emotional concerns were uttered because the patient had a need to vent her feelings. The patient’s willingness to share was in most cases interpreted as a sign of trust or good chemistry.

However, all the students explicitly or implicitly conveyed that the recorded EC-moments showed that their empathy was limited, and spent most of the time in the recall-interviews reflecting on possible reasons for this. Thus, most of the recall interviews was about what influenced the medical students’ empathy in the EC-moments. Through our analysis of the students’ perceptions, experiences, and reflections in the recall interviews, the five following key themes emerged: (1) Giving priority to medical history taking, (2) Interpreting the patient’s worry as lack of medical information, (3) Conflict between perspectives, (4) Technical communication skill rather than authentic and heart-felt and (5) The distant professional role. The results presented below are structured according to these themes. All the themes describe phenomena, tendencies or reasons that influenced their empathy in the concrete clinical encounters, and most often in a negative way. In general, key theme (2) reflects how the students’ responded to the expressed worry problematic situation and key theme (3) reflects how students responded to the frustration/anger problematic situation. Unless further specified, the findings presented below were similar for both problematic situations.

### Giving priority to medical history taking

Some students reported that their attention was primarily directed at remembering and completing the different tasks of the medical history taking and that they therefore were disrupted from or became inattentive to the patient’s ECs. This included focusing on the list of mandatory questions in a medical interview such as questions on hereditary diseases; finding out what to ask for next and covering all the different parts of the standard medical interview. Susan reported during an interview that she thought to herself:

… medical history, medical history, medical history, now suddenly I’m a doctor […] I was actually a bit preoccupied with remembering what I should ask about. And when she started bringing up the thing about the father having cystic kidney disease, it was sort of an OK transition into asking about hereditary diseases.

Sometimes, the patient’s ECs were interpreted as information relevant for further medical interviewing. James told how “the student or professional in me woke up” when the patient told of bad sleep lately related to the worry situation. As it is a typical symptom of depression, he started thinking about a scale for diagnosing depression. This distanced him a bit, he felt. He tried to do the right thing in asking about her emotional or mental health. Consequently, he felt like he dealt with the situation a bit more schematically and rationally, rather than being open and empathic, and he hoped that the patient wouldn’t notice the change in him.

### Interpreting the patient’s worry as lack of medical information

Students often reported having interpreted the patient’s emotional worry situation as a concern which could be handled with medico-professional help or advice. Students tended to think that the patient’s concern about the future was caused by a lack of medical information. Consequently, they saw it as their primary task to offer expert information or advice or offer reassurance in response to patient’s emotional worry. This interpretation of their role influenced student responses in a number of ways.

Some refrained from providing medical information or advice as a response since they felt they lacked medical competence, knowledge on prognosis, or were not yet in the proper professional role. Hannah wanted to say to the patient that she might not experience the same thing as her father but thought that she did not know enough about the disease to do so:

… how far can you go in reassuring her without doing it on false premises? so then I really just shielded myself by sitting there saying as little as possible.

Others used themselves as a reference and provided advice or reassurance the way they would have liked to receive it themselves, if they were the patient. For example, Susan both felt and consequently replied to the patient: “Maybe it will be better once they start a proper treatment and you become more aware of the situation”. Jack found an opportunity to clear up what he thought was a misunderstanding. He was uncertain whether the information the patient gave him came from her experiences with her father or was received in connection with her own condition. He wondered whether the patient really knew what it meant to have transplant surgery, and therefore felt a need to clear up her expectations in that situation and provided her with information on how not everyone with her diagnosis will need transplant surgery. Still, he did not want to go to deep into the matter since he did not feel like he had the professional competence. He said that this way she can take this information with her to her primary care physician and discuss it with him.

### Conflict between perspectives

Most students talked about how the frustration/anger situation placed them in a conflict between identifying with the patient’s perspective and that of the primary care physician.

Some students identified themselves primarily with the primary care physician. Susan remembered imagining that she was sitting there as a physician. And as a physician she could imagine that such a thing might happen in a busy professional life. She instinctively felt the need to protect the primary care physician, but shortly after realized that that was not what the patient needed. It was better to just receive the patient’s frustration instead of opposing it. Hannah was unsure and curious about whether a mistake had been made or not. She further reflected on whether she really had to know the truth to express agreement with the patient - maybe she should just agree without knowing. Mary on the other hand, was worried that the patient had already lost trust in the health care services and said during the recall interview:

I thought to myself: “[Name of communication skills teacher], what should I say now?” How should I convey that I have an understanding of what she is saying?

Consequently, she suggested to the patient: “maybe that does something to your trust relationship with your primary physician”. Later on, she reported that she is afraid to say things that sound “made up” since you contradict yourself in saying that something is sad to hear and then just move on by changing the topic of the conversation.

Other students identified more with the patient. Daniel remarked that her version did sound frustrating, but that he himself did not feel that he had enough knowledge to become angry himself. He did not feel like he could take part in frustration towards a physician he had never met and did not have a personal relationship with. Michael mentioned that he recognized the picture she was painting; he had heard similar stories before. Michael however, felt that that this was not right, it was not supposed to be that way, and that affected him. He therefore said to the patient: “that’s the sort of thing that shouldn’t happen”. Emma mentioned how she recognized the situation the patient was in from her own life. She herself had experienced how it is to have a sick father. This made her more able to understand the situation the patient was in. She added that she would have asked more about whether the patient’s experience affected her trust in the health-care system if she had more time or was her actual physician.

### Technical communication skill rather than authentic and heart-felt

When commenting on attempts to communicate understanding or interest back to the patient in the videos, students usually used technical terms to describe how they responded to the patient such as through active listening and facilitation. It was important for them to find ways to let the patient talk about her feelings and show to the patient that they indeed had understood what was being said to them. Susan said the following about her own behavior when the patient spoke of her father’s condition:

I tried to be supportive without saying too much […] to “facilitate” her a bit. I did not really say much, I mostly just nodded and said “yes”, I think. So I was kind of trying to seem understanding, yeah, professionally understanding.

According to her experience, as long as you show that you understand – even very briefly - it is ok - and she hoped that the patient saw that she listened. She adds that maybe you do not have to verbalize too much, and that often if you do that can be awkward. Later in her interview, she said that patients catch onto “fake empathy” very quickly – i.e., the physicians who do not feel any kind of empathy but still say they do. This will, according to Susan, only be attempts at empathy, but not real empathy, more like a “textbook”-form of empathy. She further added that the empathy must be real-felt- you have to feel that the person cares and understand – both emotionally and cognitively. If not, it does not matter what you say. You are supposed to try to understand the patient and want what is good for the patient - that must always be a core concern.

Emma described facilitation as a good conversational technique since you can show empathy without really feeling anything yourself. She added that there isn’t necessarily anything wrong in that, since there is no way to tell that the patient knows that you are being honest or not. She herself thought that all physicians were honest and sincere before she started medical school and learnt about conversational techniques and facilitation. Michael claimed that as a clinician you use empathy consciously as a tool to achieve something. In real life, i.e., as a “normal” fellow human, empathy is more real. He mentions that maybe you use it a bit artificially in clinical situations even though you are supposed not to. And although you might do a bit of play acting and is extra understanding to achieve something - to provide the feeling of safety or to get more information - he says it is important that it does not turn fake either.

### The distant professional role

Many students were critical of their own behavior. Students often said that they would try to show more understanding or empathy if they had the opportunity. Many students told of difficulties knowing what to say and especially what would be the right things to say as a professional, and this uncertainty seemed to result in the students being more reticent towards the patients. Susan said she found the patient’s story sad when reviewing it, but when asked if she could remember what she actually felt during the medical interview, she revealed that she entered a role – she distanced herself and did not feel the reality of it there and then.

Hannah reported that she did not know if she was allowed to ask the patient some personal questions. She was really curious to ask these questions, but was afraid they would be too personal:

I felt like I was tied up, like a coward […] I felt that, no, this is not right. But can I cross that line? […] over to the more personal and say that this is going to work out and possibly even touch her […] say things like “you seem like a strong woman”?

She chose to suppress these impulses because she felt like she had to be professional. She said that she had learnt in medical school that if you freak out, then the patient will freak out as well. You are supposed to be sensitive and empathic in a professional manner, but she did not know how, since she had never been empathic professionally before in her life.

## Discussion

The third-year medical students who took part in the present study articulated some of the difficulties related to the experience and demonstration of empathy in concrete patient encounters and shed light on what may influence medical students’ empathy when entering clinical training and practice. The students informal self-assessment of their empathy was very much in line with the results later emerging from our own detailed analysis of the interviews [24, 25]. However, this study contributes to a better and more detailed understanding of the observations made in these interviews – especially what influenced the communicative behaviors of the students.

One key finding from this study is that some of these students reported that they were primarily occupied with remembering and asking the different questions involved in recording the patient’s medical history and that this could interfere with paying attention to the patient’s emotional concerns. These same students argued that acquisition and possession of biomedical knowledge was considered more important than the emotional and relational aspects of patient encounters [20]. While these attitudes are also likely to influence students’ priorities, the results of the present study suggest that students rarely deprioritize the patient’s emotional concerns deliberately. Rather, the students are inattentive to such concerns because the students are too cognitively focused on medical history taking. Another possible reason is that the students’ horizons are shaped in a way that makes it more likely that the patient’s concerns are interpreted within a medico-professional frame of reference [9], for example when the students interpret the patients’ expressions of worry as needs for further information. Empathy also involves curiosity about another’s distinct experience [29], and it has been claimed that the natural curiosity with which students enter medical school, atrophies as they become gradually more assimilated within medical culture [30]. When attempting to accommodate to implicit or explicit ideals of medical history taking, students can miss important aspects, for example how the illness affects this individual patient [31] and the individual patient’s needs and preferences.

Although these students described recognizing and understanding the patients’ emotions, they only occasionally experienced empathic concern for the patient. In general, the students’ understanding both reflected and was more consistent with the ideal of cognitive empathy which is generally encouraged within medical education [7, 32]. This more objectivistic form of empathy is closely related to the idea that it is possible and advisable to understand the patient’s perspective without being affected emotionally and without bias [9, 32] and to ideals of detached concern [17], affective neutrality [33] and a more general form of objectivism that have been reported to be present in medical schools [34]. The empathic experiences reported by the students may very well reflect a transitory phase as they adapt to their recently acquired professional identities [15, 20]. However, other studies suggest that students will not make use of later opportunities in their careers to develop alternative ways of communicating, but will continue to respond to patient emotion with biomedical questioning, information giving, nonspecific acknowledgement or premature reassurance [35–41]. For example, Agledahl et al. demonstrated that physicians working in hospitals mask a neglect of patients’ existential worries with politeness [42]. These physicians actively directed focus away from patients’ existential concerns, focused more on medical facts and rarely addressed personal aspects of patients’ situations.

While empathy was generally regarded as important and appropriate in the situation, some students struggled with combining empathy and compassion with professional norms and ideals. While the recent addition of communication skills training to the medical curriculum certainly has put empathy on the agenda, some students seem to regard rules for professional communication with patients as absolutes. It is possible that communication skills courses may contribute to this uncertainty by providing the illusion that there is always a professionally correct way to respond or communicate. If the institutional role in which students find themselves permits little or no space for the expression of their own emotional reactions, interpretations and judgments, the very format of the medical interview may contribute to reduce awareness of or even extinguish students’ affective responses and expressions of own interpretations. Roter and Hall claim that roles in provider-patient relationships are just a kind of conformity, not moral codes or rule of law [43]. Our results nuance this claim in that implicit or explicit ideals for medical interviews and professional empathy can be perceived as guiding principles of conduct as well as rules for professional or right empathic behavior.

Larson and Yao compare the physician’s role in empathic interaction to that of an actor [44]. They further argue that there are no apparent ethical issues in this because we as human beings hide our true feelings all the time. We would however argue that the discrepancies seen between students’ instrumental ways of providing understanding to patients (such as the application of skills or techniques to let the patient vent her feelings) and their own more personal or lay norms of empathy indeed constitutes a moral dilemma. We find it worrisome that students are sometimes taught to perform forms of play-acting to convey that they understand the patient’s emotional issues regardless of what the students are actually thinking and feeling. By separating instrumental outcome-focused empathic behavior from the broader interpretive, interpersonal, moral, existential, and emotional dimensions of empathy, important aspects and relations in clinical perceptions and judgment may be lost. The main focus of students seems to be on the parts of the patients’ narrative that the students can act on as physicians and not to what they can respond to as fellow humans. If medical students are mainly encouraged to perform medical tasks effectively and not meet patients as fellow human beings, core aspects of the students future role as physicians seem to be challenged at an already early point in their careers [45].

A strength of this study is the use of detailed, in-depth video recall-interviews to investigate students’ experiences and reflections on their own empathic behavior through qualitative inquiry rather than statistical data. These findings provides crucial knowledge about the motivations and considerations behind the communicative behavior of the eleven students and are also supported by semi-quantified observational data published from the same larger study [24, 25]. To our knowledge, this study is the first of its kind. As far as we know, no significant changes have been made to the curriculum in communication skills teaching at this particular institution since the data were collected. Due to the department responsible for the study, the participating students were probably more than averagely interested in empathy and communication. Our hopes for the future are that the present study can encourage scholars to conduct studies with innovative approaches, designs and multiple methods in order to study the complex phenomenon of empathy in clinical settings. We would also like to invite medical educators and practitioners to encourage student reflection and discussion on how to conduct a medical history with curiosity for both the patient’s as well as their own thoughts, feelings, interpretations and perspectives.

## Conclusion

In this qualitative analysis, five themes which may influence student empathy during history-taking were identified: (1) Giving priority to medical history taking, (2) Interpreting the patient’s worry as lack of medical information, (3) Conflict between perspectives, (4) Technical communication skill rather than authentic and heart-felt and (5) The distant professional role. To our knowledge, this is the first study ever to document the students’ own perspective in concrete situations as well as how these reported experiences and reflections affect their empathy towards patients. Since we now know more about what is likely to hinder medical students’ empathy, educators should actively encourage group reflection and discussion in order to avoid the negative effects of history taking both inside and outside of the clinical setting.

## Data Availability

The datasets used and/or analyzed during the current study are available from the corresponding author on reasonable request.

## Abbreviations

SP: Simulated patient
EC: Emotional Concern
